# 503 Should Co-morbid Conditions Be Factored in Mortality Prediction Models for Burn Injury: Single Center Evaluation

**DOI:** 10.1093/jbcr/irae036.138

**Published:** 2024-04-17

**Authors:** Jamie L Hollowell, Ashley Levine, Benjamin Bodek, Chris B Agala, Eli Maxwell, Lori Chrisco, Robert W Matthews, booker King, Felicia Williams

**Affiliations:** University of North Carolina, Chapel Hill, NC; University of North Carolina, Durham, NC; University of North Carolina Chapel Hill, carrboro, NC; UNC Chapel Hill - School of Medicine, Surgery dept, Chapel Hill, NC; UNC Chapel Hill Jaycee Burn Center, Chapel Hill, NC; NC Jaycee Burn Center, Chapel Hill, NC; UNC Chapel Hill. NC, Chapel Hill, NC; The University of North Carolina at Chapel Hill, Chapel Hill, NC; University of North Carolina, Chapel Hill, NC; University of North Carolina, Durham, NC; University of North Carolina Chapel Hill, carrboro, NC; UNC Chapel Hill - School of Medicine, Surgery dept, Chapel Hill, NC; UNC Chapel Hill Jaycee Burn Center, Chapel Hill, NC; NC Jaycee Burn Center, Chapel Hill, NC; UNC Chapel Hill. NC, Chapel Hill, NC; The University of North Carolina at Chapel Hill, Chapel Hill, NC; University of North Carolina, Chapel Hill, NC; University of North Carolina, Durham, NC; University of North Carolina Chapel Hill, carrboro, NC; UNC Chapel Hill - School of Medicine, Surgery dept, Chapel Hill, NC; UNC Chapel Hill Jaycee Burn Center, Chapel Hill, NC; NC Jaycee Burn Center, Chapel Hill, NC; UNC Chapel Hill. NC, Chapel Hill, NC; The University of North Carolina at Chapel Hill, Chapel Hill, NC; University of North Carolina, Chapel Hill, NC; University of North Carolina, Durham, NC; University of North Carolina Chapel Hill, carrboro, NC; UNC Chapel Hill - School of Medicine, Surgery dept, Chapel Hill, NC; UNC Chapel Hill Jaycee Burn Center, Chapel Hill, NC; NC Jaycee Burn Center, Chapel Hill, NC; UNC Chapel Hill. NC, Chapel Hill, NC; The University of North Carolina at Chapel Hill, Chapel Hill, NC; University of North Carolina, Chapel Hill, NC; University of North Carolina, Durham, NC; University of North Carolina Chapel Hill, carrboro, NC; UNC Chapel Hill - School of Medicine, Surgery dept, Chapel Hill, NC; UNC Chapel Hill Jaycee Burn Center, Chapel Hill, NC; NC Jaycee Burn Center, Chapel Hill, NC; UNC Chapel Hill. NC, Chapel Hill, NC; The University of North Carolina at Chapel Hill, Chapel Hill, NC; University of North Carolina, Chapel Hill, NC; University of North Carolina, Durham, NC; University of North Carolina Chapel Hill, carrboro, NC; UNC Chapel Hill - School of Medicine, Surgery dept, Chapel Hill, NC; UNC Chapel Hill Jaycee Burn Center, Chapel Hill, NC; NC Jaycee Burn Center, Chapel Hill, NC; UNC Chapel Hill. NC, Chapel Hill, NC; The University of North Carolina at Chapel Hill, Chapel Hill, NC; University of North Carolina, Chapel Hill, NC; University of North Carolina, Durham, NC; University of North Carolina Chapel Hill, carrboro, NC; UNC Chapel Hill - School of Medicine, Surgery dept, Chapel Hill, NC; UNC Chapel Hill Jaycee Burn Center, Chapel Hill, NC; NC Jaycee Burn Center, Chapel Hill, NC; UNC Chapel Hill. NC, Chapel Hill, NC; The University of North Carolina at Chapel Hill, Chapel Hill, NC; University of North Carolina, Chapel Hill, NC; University of North Carolina, Durham, NC; University of North Carolina Chapel Hill, carrboro, NC; UNC Chapel Hill - School of Medicine, Surgery dept, Chapel Hill, NC; UNC Chapel Hill Jaycee Burn Center, Chapel Hill, NC; NC Jaycee Burn Center, Chapel Hill, NC; UNC Chapel Hill. NC, Chapel Hill, NC; The University of North Carolina at Chapel Hill, Chapel Hill, NC; University of North Carolina, Chapel Hill, NC; University of North Carolina, Durham, NC; University of North Carolina Chapel Hill, carrboro, NC; UNC Chapel Hill - School of Medicine, Surgery dept, Chapel Hill, NC; UNC Chapel Hill Jaycee Burn Center, Chapel Hill, NC; NC Jaycee Burn Center, Chapel Hill, NC; UNC Chapel Hill. NC, Chapel Hill, NC; The University of North Carolina at Chapel Hill, Chapel Hill, NC

## Abstract

**Introduction:**

Comprehensive conversations about prognosis in burns currently neglect the impact of co-morbid conditions on outcomes. Currently, the most often quoted burn mortality predictors focus on age, burn size and depth, and inhalation injury. We suspect comorbidities increase the risk of mortality from a large burn. Our aim is to identify and quantify the magnitude of impact pre-existing comorbidities may have on the outcomes of our burn patients.

**Methods:**

Patients were identified using Institutional Burn Center registry and linked to the clinical and administrative data. Adult patients admitted from Jan 1, 2012, to June 30, 2023, were included. Demographics, length of stay (LOS), and mortality were evaluated using logistic regression to create a predictive model. LASSO regularization was used for variable selection; model was fit with a training dataset (n=7948) and evaluated on a validation dataset (n=1975).

**Results:**

We evaluated 23 variables to identify variables with independent predictive power. Fourteen variables had increased predicted risk of mortality: female sex (0.07), increasing age (0.065), etiology other than burn(0.36), burn size (0.8), public insurance (0.27), inhalation injury (1.92), positive alcohol status upon admission (0.07), COPD (0.85), congestive heart failure (0.56), stroke (0.36), diabetes (0.08), renal failure (0.97), alcoholism (0.64), and substance abuse (0.51). Conversely, 5 variables led to a decreased predicted risk of mortality: other/unknown insurance status (-0.23), unknown alcohol history (-0.24), positive cocaine status upon admission (-0.22), asthma (-0.18), and hypertension (0.24). The model’s AUC on the validation dataset was 0.926.

**Conclusions:**

Not all co-morbid conditions effect outcomes negatively. Additionally, the lack of association of some variables may be attributed to the presence of other comorbidities accounting for their impact. Next steps are to determine if any of these factors are modifiable with changes in treatment strategies regardless of the chronicity of the co-morbid condition.

**Applicability of Research to Practice:**

Current burn mortality predictors lack many comorbid factors, so further work needs to be done to determine if additional comorbid conditions should be considered in formal mortality predictions.

